# Bitumen-Based Poroelastic Pavements: Successful Improvements and Remaining Issues

**DOI:** 10.3390/ma16030983

**Published:** 2023-01-20

**Authors:** Piotr Jaskula, Jerzy A. Ejsmont, Wladyslaw Gardziejczyk, Piotr Mioduszewski, Marcin Stienss, Marek Motylewicz, Cezary Szydlowski, Pawel Gierasimiuk, Dawid Rys, Marta Wasilewska

**Affiliations:** 1 Department of Transportation Engineering, Faculty of Civil and Environmental Engineering, Gdansk University of Technology, 11/12 Gabriela Narutowicza St., 80-233 Gdansk, Poland; 2 Automotive and Military Technology Division, Faculty of Mechanical Engineering and Ship Technology, Gdansk University of Technology, 11/12 Gabriela Narutowicza St., 80-233 Gdansk, Poland; 3 Division of Road Engineering, Faculty of Civil Engineering and Environmental Sciences, Bialystok University of Technology, 45E Wiejska St., 15-351 Bialystok, Poland

**Keywords:** highly polymer-modified bitumen, pavement fire-fighting properties, poroelastic asphalt mixture, rolling resistance, tyre/road noise, water permeability

## Abstract

This article presents the development process of designing and testing poroelastic pavement based on highly polymer-modified bitumen. Poroelastic wearing course was composed of mineral and rubber aggregate mixed with highly polymer-modified bitumen, in contrast to previous trials, during which polyurethane resins were mainly used as binder, which led to several serious technological problems concerning difficult production, insufficient bonding to the base layer, and unsatisfactory durability. The laboratory testing phase was aimed at finding the proper composition of the poroelastic mixture that would ensure required internal shear strength and proper bonding of the poroelastic layer with the base layer. After selecting several promising poroelastic mixture compositions, field test sections were constructed and tested in terms of noise reduction, rolling resistance and interlayer bonding. Despite the very good acoustic properties of the constructed poroelastic wearing course, it was not possible to solve the problem of its insufficient durability. Still, the second major issue of poroelastic pavements that concerns premature debonding of the poroelastic layer from the base layer was completely solved. Experience gained during the implementation of the described research will be the basis for further attempts to develop a successive poroelastic mixture in the future.

## 1. Introduction

Interaction between vehicle tyre and road surface belongs to crucial factors that affect road safety, ride quality, and energy consumption. Currently, the most important parameters regarding the interaction of the tyre with the road were defined in the system of labels describing the quality of car tyres. These are: grip, noise, and rolling resistance. These parameters depend on both the tyre itself and the road surface. This article presents activities that are related to the creation of an “ultra-quiet” road surface, which contributes to a significant reduction in tyre/road noise.

In order to obtain the reduction, it is necessary to simultaneously inhibit all tyre noise generation mechanisms, i.e., mechanisms related to air flow and the impact of tread elements on the pavement surface [1]. The noise generation efficiency of aerodynamic mechanisms can be significantly reduced by good ventilation of the tyre contact area through a system of interconnected air channels in the surface, i.e., owing to the porosity of the surface. Reduction in the effectiveness of impact mechanisms can be achieved through a significant reduction in the stiffness of the pavement and introduction of materials with significant energy dissipation properties. Pavements that can potentially be porous and flexible at the same time are called poroelastic pavements and are characterised by an open pore structure and a significant proportion of rubber aggregate ensuring flexibility.

Work on such surfaces was initiated in Sweden in the 1970s and is still ongoing worldwide due to numerous problems that emerged during development. Over the last 30 years, various variants of poroelastic pavements were investigated (see Table 1). Tests were made on surfaces containing polyurethane and bitumen binders (including highly modified bitumen). Tests were performed on materials in which all aggregate came from tyre recycling and materials containing greater or lesser quantities of fine mineral aggregate, allowing for a significant improvement in skid resistance, because pavements without such aggregate displayed serious problems with wet friction despite good drainage properties of the material.

After over three decades of research work it can be said that the most difficult and still unsolved problem regarding poroelastic pavements is their insufficient durability [9,15,16]. Such wearing courses are characterised by very good acoustic properties (noise reduction above 10 dB compared to the typical, most popular pavements), sufficiently good skid resistance (in the case of black ice, even much better than classic surfaces) and, unfortunately, relatively high rolling resistance. Very specific features of poroelastic pavements include their excellent fire-fighting properties, as they suppress the spread of fuel spill fires very effectively, which makes them suitable for use in tunnels and other dangerous locations. Thus, in order to enable usage of these pavements on public roads, it is necessary to solve the problem of their insufficient durability, which can be caused by insufficient interlayer bonding of the poroelastic layer to the binder/base course or insufficient internal integrity of the poroelastic layer itself.

## 2. Objectives

This article presents the results of the “Safe and Eco-friendly Poroelastic Road Surface” (SEPOR) research project, the main goal of which was to evaluate the possibility of producing a poroelastic mixture using highly polymer-modified bitumen, instead of the polyurethane binder that was commonly used by other researchers. Such a change was desirable from the technological point of view. It should be pointed out that mixtures based on polyurethane binder are very difficult in terms of production and laying technology, because they require specific thermal and humidity conditions. Moreover, as the stiffness of the polyurethane binder is considerably higher than that of bitumen, it induces high stresses between the poroelastic layer and the layer underneath, which is usually made of traditional asphalt mixture. Replacement of polyurethane binder with highly polymer-modified bitumen in a poroelastic mixture could potentially resolve these issues and still maintain very good acoustic properties, acceptable skid resistance and rolling resistance, and decrease fire hazard. It should also be noted that the price of polyurethane binder is much higher than the price of highly polymer-modified bitumen.

## 3. Research Methodology

The main problems to be solved during the design stage of the composition of the poroelastic mixtures were: selection of asphalt binder, selection of mineral mixture components and size of rubber granulate, selection of the type of mixture due to the type and maximum grain size, development of technology for bonding the poroelastic layer with the layer underneath, and development of technology for laying and compacting of the poroelastic layer. 

**Figure 1:**
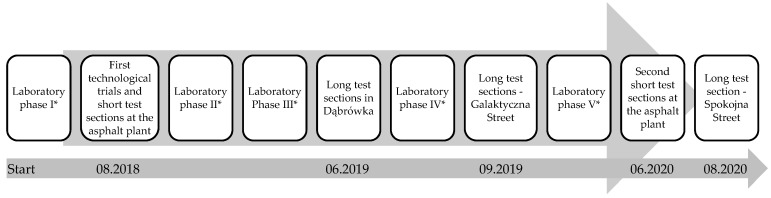
Scheme of the development of the SEPOR poroelastic mix; * detailed description of laboratory phases is provided in Section 3.2.

To achieve the goals described above, the design work consisted of continuous verification of the technologies developed in the laboratory through real scale production and construction of field test sections. The development of the poroelastic mixture design process is presented in Figure 1.

### 3.1. Materials Used

The main assumption of the SEPOR project was to obtain a poroelastic mixture using asphalt binder instead of polyurethane epoxy resins. Three types of bitumen were selected for preliminary tests: one standard SBS-modified bitumen 10/40–65 (approximately 3% content of SBS by mass of bitumen) and two highly SBS-modified bitumens (approximately 7.5% content of SBS by mass of bitumen): 45/80–80 and 65/105–80. These bitumens were produced in a refinery and applied in the research as a final product. The properties of bitumens used are shown in Table 2. 

**Figure 2:**
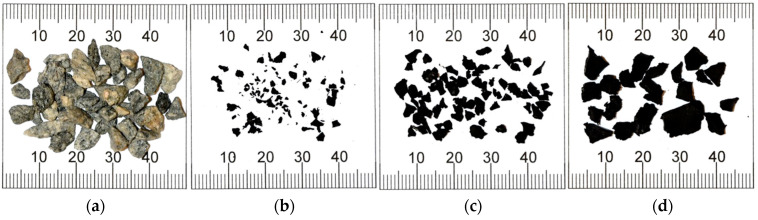
Mineral and crumb rubber materials used in the tests: (**a**) gneiss coarse aggregate 2/5; (**b**) crumb rubber 0.5/2; (**c**) crumb rubber 1/4, and (**d**) crumb rubber 4/7.

The mineral part of the poroelastic mixture contained coarse crushed gneiss aggregate (2/5, 4/8, 5/11, and 11/16), fine gneiss aggregate, and limestone filler. The rubber part of the mixture was composed of crumb rubber obtained through the tyre recycling process. The recycling process used a shredding technique at ambient temperature. Different fractions of crumb rubber were used in this project: 0.5/2, 1/4, and 4/7 mm. Coarse crushed gneiss aggregate of the 2/5 mm fraction and crumb rubber of different sizes are shown in Figure 2.

**Figure 3:**
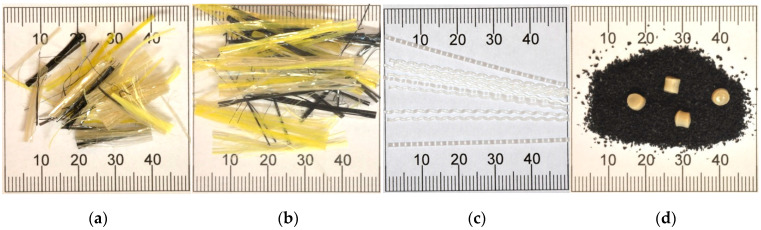
The additives used in the tests: (**a**) 19-mm-long aramid fibres; (**b**) 38-mm-long aramid fibres; (**c**) 50-mm-long polymer fibres, and (**d**) crumb rubber and polyoctynamer polymer modification applied to bitumen.

During the initial stage of laboratory testing, two additional modifications of the production process were also tested: using polymer fibres as reinforcement and additional modification of bitumen with crumb rubber. Two types of fibres were investigated. One type was aramid-polyalphaolefin fibre, which is produced specifically for use as reinforcement in asphalt mixtures. In this research two lengths of these fibres were used: 19 mm and 38 mm. The fibres were added at the quantity of 0.05% by weight of asphalt mixture. The second type of fibre was 50-mm-long polymer fibre, used normally as reinforcement for cement concrete mixtures, at the quantity of 0.05% by weight of asphalt mixture. An additional crumb rubber modification method was conducted in the laboratory using a special crumb rubber additive technology. This process consisted of mixing 191 g of ground tyre rubber (0.2/0.8 mm), 9 g of a specific polymer (polyoctynamer), and 2000 g of asphalt binder. The mixing process was performed using laboratory mixer at 200 RPM for 120 min. Temperature during the mixing process was kept within the range of 170–180 °C. Different additives used in the tests are shown in Figure 3.

### 3.2. Granular Mixture Design Evolution

The design of an adequate poroelastic mixture composition was divided into five major phases, reflecting the successive steps of selection and optimisation. The prefix “P” before the mixture type abbreviation means “poroelastic”. During these phases, many different variants of poroelastic mixtures were tested. The mineral skeleton of these mixtures was based on traditional asphalt mixtures: SMA 5 (stone mastic asphalt with a maximum grain size of 5 mm), SMA 8, SMA 11, PA 8 (porous asphalt with a maximum grain size of 8 mm), PA 11, MNU 8 (gap-graded asphalt mixture with a maximum grain size of 8 mm), and MNU 11. The composition of these mixtures (see Table 3) was based on Polish and Swedish technical regulations concerning mineral gradation of asphalt mixtures.

During the first phase, the mix design was based on the SMA 5 mixture that was used during the PERSUADE research program [17,18,19]. The main objective of the first phase of the research was to select the best asphalt binder and to assess the influence of different additives and production techniques on the properties of the investigated mixtures. For instance, apart from the aforementioned additives, three different ways of adding the asphalt binder to the mineral–rubber mixture were also tested: (1) adding the asphalt binder into the mixer directly onto the hot aggregate; (2) initial addition of the asphalt binder onto the crumb rubber only, storing this pre-mixture at 160 °C for 120 min and, only after that, adding it to the hot aggregate; (3) initial wet modification of the asphalt binder with fine crumb rubber and adding it to the mineral–rubber aggregate. A total of nine different mixtures were tested at this stage: two reference mixtures (only with stone aggregate) and seven poroelastic ones (with 20% addition of crumb rubber).

Due to the fact that the poroelastic mixtures from the first phase showed inadequate resistance to permanent deformation, research efforts in the second phase were focused on finding the maximum amount of crumb rubber that could be added to the mixture without significant impact on its durability and resistance to wheel tracking test. As a result, different base gradation curves (SMA 8 and MNU 8) were selected. Additionally, at this stage, two different fractions of crumb rubber (0.5/2 and 1/4) were mainly used (still, only one fraction of crumb rubber was used in a given mixture). In total, 20 different combinations of mixture design were tested at this stage: 2 reference mixtures (only with stone aggregate) and 18 poroelastic ones.

The third phase of the research reflected conclusions drawn from the second stage of the research, which clearly indicated that higher resistance to permanent deformation can be achieved by decreasing air void content (thus also negatively affecting the texture of the poroelastic layer and acoustic properties). Due to significant difference in density between the aggregate (2.5–2.8 g/cm^3^) and crumb rubber (1.0 g/cm^3^), an altogether different methodology of designing mineral–crumb rubber mixture was adopted based on the volume of each component. The main objective of this research phase was to design a mixture with: (1) air void content between 15% and 20%, and (2) internal shear strength at the level of 1 MPa. At this stage, neat road bitumen was also tested for the first time, to highlight the pure influence of mineral–rubber mixture on the properties of the future poroelastic layer. The second asphalt binder that was tested was highly polymer–modified bitumen 45/80–80.

For the fourth phase of the mix design research, selected PSMA 5 and PSMA 8 mixtures that achieved the best results in the previous phase were chosen for further testing. For these mixtures, minor changes in mastic composition were introduced, including (1) reduction in limestone filler content with an increase in asphalt binder content, (2) simultaneous increase in both limestone filler content and asphalt binder content.

During the fifth phase of laboratory mix design research, the addition of hydrated lime was applied and investigated to improve the affinity of binder and aggregate. The aim of the changes was to design a more open mix with larger pore diameter in order to improve the water permeability and acoustic properties of the layer. This goal was achieved by using mineral aggregate 4/5.6 instead of 2/5.6, which necessitated an additional process of aggregate screening before using it in the mix. In addition, single-fraction rubber grit 1/4 was replaced back to a 2/4 fraction. The amount of lime filler was also reduced. Warm mix additive Sasobit was also used in one variant to verify whether such an additive would improve workability and compactibility of the poroelastic mixture. 

### 3.3. Laboratory Test Methods

Due to the lack of any available guidelines or requirements concerning the design of poroelastic mixtures, several different tests were adopted to assess their internal cohesion and durability. The summary of test methods is presented in Table 4. After preliminary trials, it was established that two parameters would be taken into consideration in the initial phase of the selection process: (1) air void content, and (2) internal shear strength of the layer obtained from the Leutner apparatus. It was assumed that the internal shear strength of the layer is mainly responsible for the durability of the mixture in the field. Only mixtures that achieved the target levels of these two parameters (air voids content >15%, internal shear strength ~1 MPa @+20 °C) were chosen for further testing, including a wheel tracking test. Some of the tests had to be discontinued because of their inadequate and unreliable results. For example, the loss of mass of poroelastic samples during the Cantabro test (according to the EN 12697-17 standard) was minimal, because of their elasticity and tendency to easily bounce inside the Los Angeles device.

### 3.4. Laboratory Test Results

During all five stages of selection of the poroelastic mix composition, about one hundred twenty variants were tested, differing in mineral mix, content and fractions of rubber, content and type of asphalt binder, and type of additives (reinforcing fibres, WMA additives, and hydrated lime additive). In total, with the addition of samples taken from the test sections that differed in the method of compaction and type of interlayer bonding technique, over 200 variants were tested [11,12,20,21,22,23,24,25].

Table 5 presents selected results from the research. Because of the vast range of tested mixtures, only selected results are presented. 

Summarising the test results of all the evaluated variants, it can be concluded that:

- void content increases with an increase in rubber granulate content,
- void content decreases with an increase in bitumen content,
- shear strength decreases with an increase in rubber granulate content,
- and shear strength increases with bitumen content.

**Figure 4:**
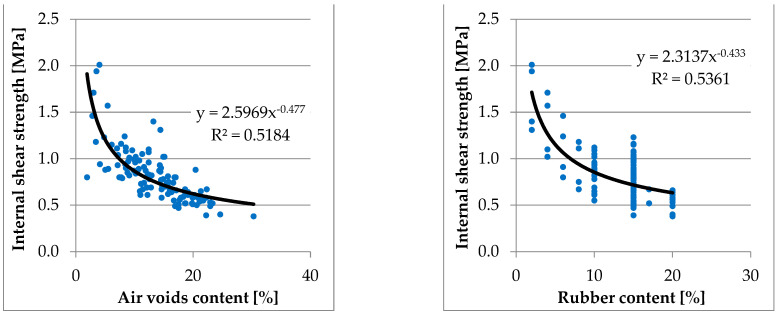
Summary of laboratory test result of internal shear strength versus air voids and rubber content.

Global dependencies are shown in Figure 4.

It was concluded that maintaining the desired level of air voids above 15% and the content of rubber granulate above 15% results in a shear strength of 0.5 and 1 MPa. 

### 3.5. Interlayer Bonding

The second major issue to be solved during the SEPOR project was the risk of potential delamination of the poroelastic layer from the layer located underneath. Such pavement distress was observed very often during previous studies and research programs. Several different bonding techniques were evaluated [26], including laying of the SEPOR mixture over a milled bituminous layer with coarse grooves [27]. Bonding quality was assessed using the direct shear test under monotonic load and the cyclic direct shear test in the advanced shear tester (AST) device [23].

**Figure 5:**
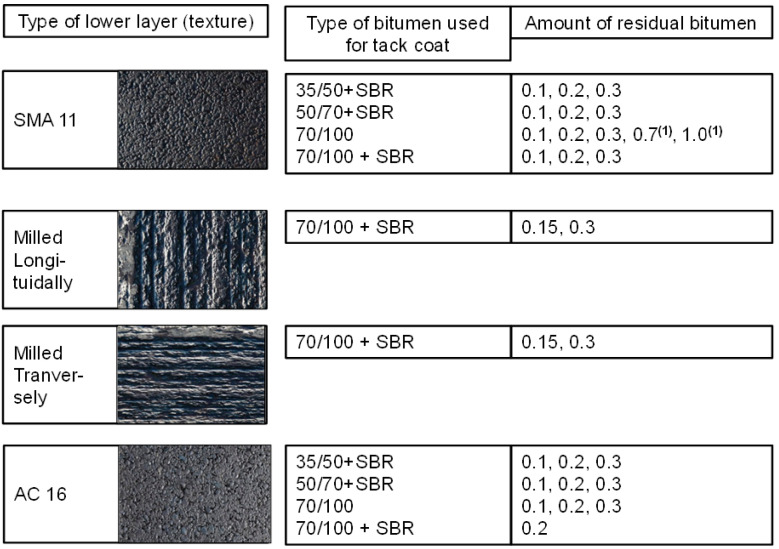
Combinations of bonding techniques used in the project; ^(1)^ hot bitumen instead of emulsion.

At the first stage of interlayer bonding laboratory testing, over a dozen different interlayer combinations were tested. These combinations are shown in Figure 5. Based on the laboratory test results, interface variants for further testing on full-scale test sections were chosen.

Two types of asphalt mixture for the lower layer were analysed: asphalt concrete AC 16 W with 35/50 road bitumen (with a standard smooth surface texture typically obtained after compaction with drum roller compactor) and stone mastic asphalt SMA 11 with PMB 45/80-55 modified bitumen, with various surface texture combinations: standard smooth, grooved longitudinally or transversely (due to milling) as well as milled and covered with carbon/glass fibre reinforcement.

Tack coat was applied by spraying the interface with the following types of bituminous materials:

- bituminous emulsion C60B3 70/100,
- bituminous emulsion C60BP3 70/100 with SBR polymer,
- bituminous emulsion C60BP3 50/70 with SBR polymer,
- bituminous emulsion C60BP3 35/50 with SBR polymer,
- and hot bitumen 70/100.

**Figure 6:**
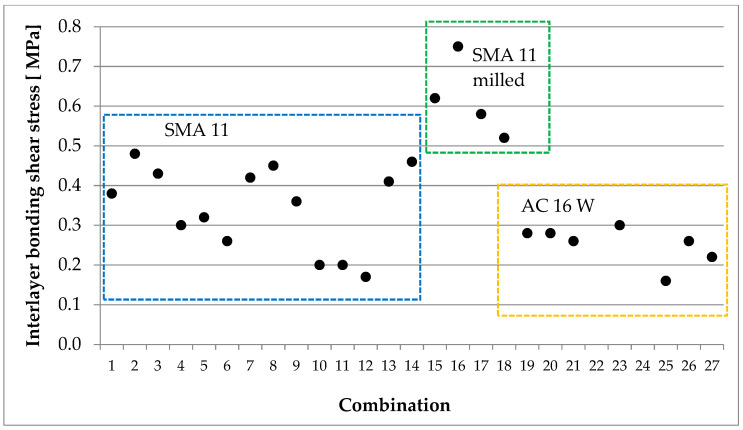
Interlayer bonding test result—shear stress.

Application rates of the bituminous emulsions were set in order to obtain the following amounts of residual bitumen on the treated surface: 0.1, 0.2, or 0.3 kg/m^2^ for specimens without grooves and 0.15 or 0.3 kg/m^2^ for specimens with grooves. All emulsions were dedicated for interlayer tack coat. For application of hot bitumen, amounts of 0.7 and 1.0 kg/m^2^ (that cannot be applied with the use of asphalt emulsion) were taken into account to verify the impact of a higher amount of binder on bond quality. Moreover, at full-scale test sections the influence of a pre-bituminised carbon/glass fibre grid placed at the interface was also evaluated. The function of the grid was to reinforce the bottom of the poroelastic layer. A more detailed description of the sample preparation process for interlayer bonding testing is presented in [21]. In total, specimens representing 27 combinations of layer interfaces were tested in the preliminary laboratory tests. For each combination, two specimens were tested. A summary of the test result is presented in Figure 6.

Based on the tests carried out on samples prepared in the laboratory, it was established that milling of the upper surface of the binder layer (irrespectively of the mixture type being used–asphalt concrete or stone mastic asphalt) must be applied during construction of full-scale test sections to achieve proper interlayer bonding strength.

### 3.6. Full-Scale Production

After the laboratory phase, selected mixtures were chosen for the first experimental field application. The purpose of this trial was to assess the possibility of using an ordinary asphalt batch plant, paver, and compacting equipment for production, laying, and compaction of poroelastic mixtures, with particular focus on:

- adding crumb rubber to the pugmill of the asphalt batch plant by means of belt conveyor normally used for adding reclaimed asphalt pavement (RAP),
- mixing of the poroelastic mixture in ordinary pugmill of the asphalt plant,
- using two ordinary asphalt pavers (one for normal carriageways, and smaller one for bicycle paths) for placing the layer of poroelastic mixture,
- and applying different techniques of compaction, i.e., light and heavy rollers, both with and without vibration mode.

First full-scale production test proved that it is possible to produce and lay poroelastic mixture with asphalt binder using conventional road construction equipment, with minor adjustments concerning the following issues:

- using a conveyor belt normally used for adding RAP for feeding rubber aggregate; in spite of windy conditions during production, crumb rubber was not blown off the conveyor belt and it was possible to precisely dose the required amount of rubber;
- mixing time and temperature should be altered to take into consideration the ambient temperature of rubber aggregate that is added directly into the pugmill of the asphalt plant;
- and paver screed should be hydraulically controlled (not only by the weight of the screed itself) to maintain proper thickness of the poroelastic layer.

### 3.7. Full-Scale Test Sections

During the project, two series of short test sections and three full-scale (long) test sections were constructed. Short test sections were located directly on the asphalt plant site (MTM). One full-scale test section was constructed on a road on private property in Dąbrówka, the other two were located on public roads: Galaktyczna St. in Gdańsk and Spokojna St. in Kartoszyno.

Technological trials and test sections were carried out in the following chronology:

- first short test section (MTM1) described in Section 3.6—asphalt plant, September 2018,
- first long test section (D)—Dąbrówka, June 2019,
- second long test section (G)—Galaktyczna St., Gdańsk, September 2019,
- second short test section (MTM2)—asphalt plant, June 2020,
- and third long test section (K)—Spokojna St., Kartoszyno, September 2020.

Construction of the section in Dąbrówka and Galaktyczna Street in Gdańsk is presented in detail in [24]. Detailed information about second short test section is given in [25]. In the case of the third long test section (K), the selection of poroelastic mixtures to be used was based on additional tests from the second short test sections constructed in 2020 (MTM2). Mixtures chosen for the final, longest test section (K) differed from those previously used in Dąbrówka and Galaktyczna St. in Gdańsk in the following characteristics:

- mineral aggregates with shortened fractions were used (screened coarse aggregates),
- single-fraction rubber aggregate was used,
- an addition of hydrated lime was used,
- and special asphalt binder was used, with appropriately composed chemical additives to promote adhesion between binder and rubber aggregate.

Ultimately, the following mixtures were selected to be used on the final, longest test section in Kartoszyno (K) (parameters given in Table 4), which was divided into four sectors that differed in terms of mixture used and interlayer bonding technique:

- PSMA5 W7 HL CR15 A10 (sector 1 and 2).
- PSMA8 W9 HL CR4/5.6 15 A10 (sector 3 and 4).

Owing to the performed optimisation of the interlayer bonding of the poroelastic mixture with the underlying layer, and based on experience from sections in Dąbrówka and Galaktyczna St., two bonding techniques were selected:

- longitudinal milling of the binder layer made of stone mastic SMA 11 45/80-55 with application of modified asphalt emulsion for interlayer bonding C 60 BP3 ZM (sector 1 and 3),
- and application of modified asphalt emulsion for interlayer bonding C 60 BP3 ZM on the binder layer made of porous asphalt PA 11 45/80-55 (sector 2 and 4).

**Figure 7:**
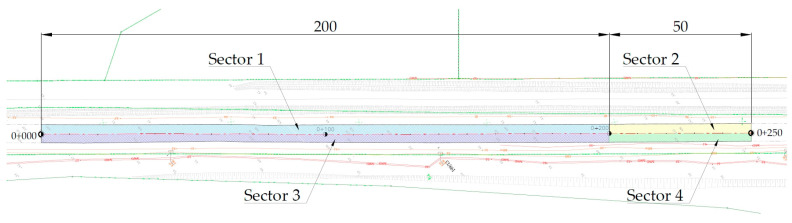
General layout of the Kartoszyno long test section.

Sectors without longitudinal milling were tack-coated with bitumen emulsion with a dosage rate of 0.2 kg/m^2^ (bitumen residue), while on sectors with longitudinal milling, tack coat dosage rate was increased to 0.3 kg/m^2^ (bitumen residue). The general layout of the Kartoszyno test section is shown in Figure 7.

**Figure 8:**
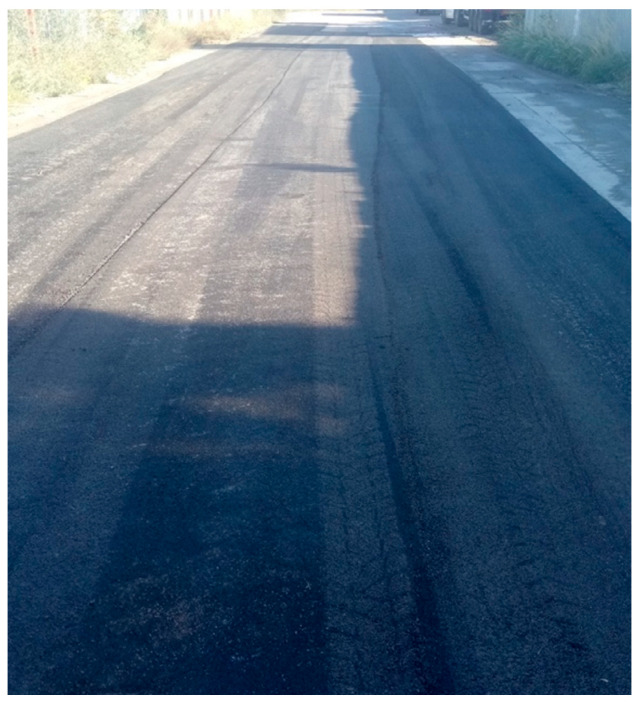
First short test section at the asphalt plant (MTM1) constructed during technological trials described in Section 3.6—September 2018.

**Figure 9:**
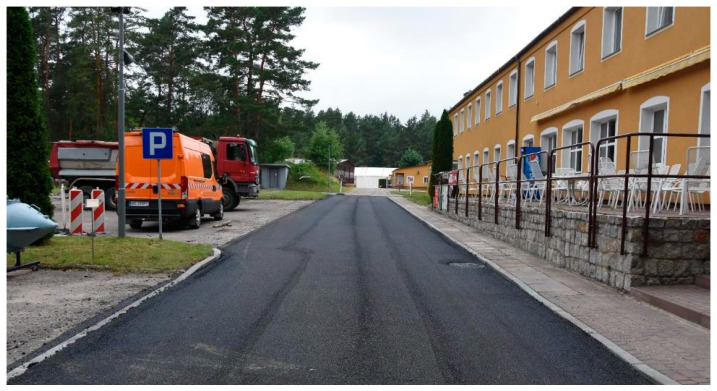
First long test section (D)—Dąbrówka, June 2019.

**Figure 10:**
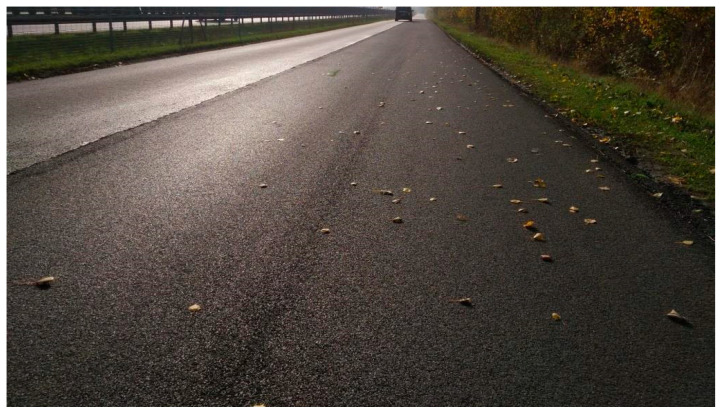
Second long test section (G)—Galaktyczna St., Gdańsk, September 2019.

**Figure 11:**
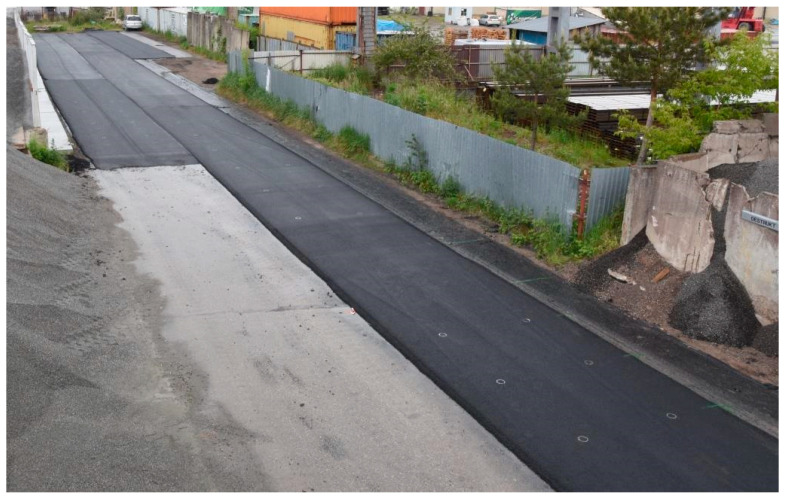
Second short test section located at the asphalt plant (MTM2)—June 2020.

**Figure 12:**
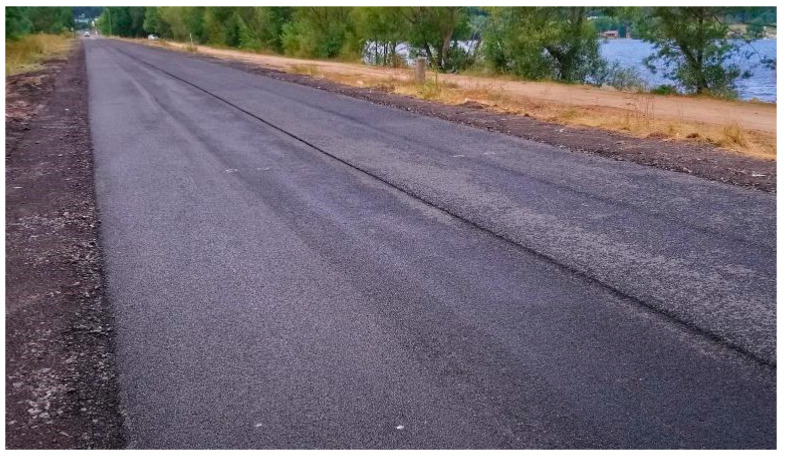
Third, final long test section (K)—Spokojna St., Kartoszyno, September 2020.

Figure 8, Figure 9, Figure 10, Figure 11 and Figure 12 present general views of various test sections.

**Figure 13:**
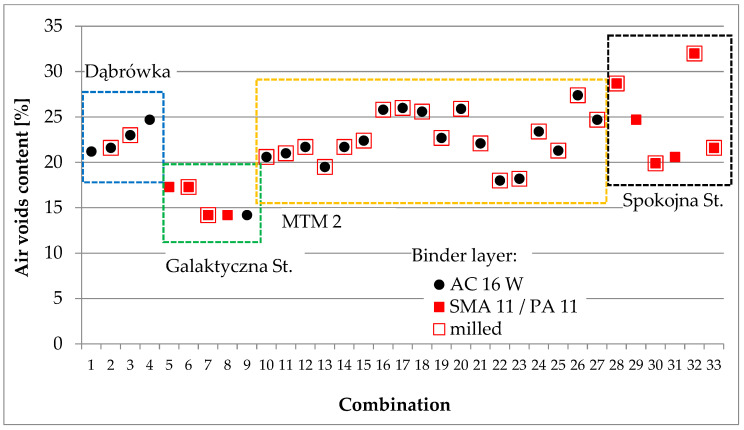
Summary of air voids content results for samples from test sections.

**Figure 14:**
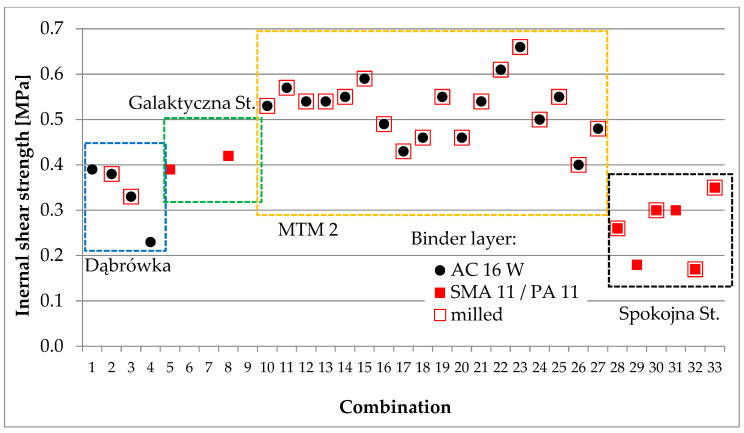
Summary of internal shear strength results for samples from test sections.

**Figure 15:**
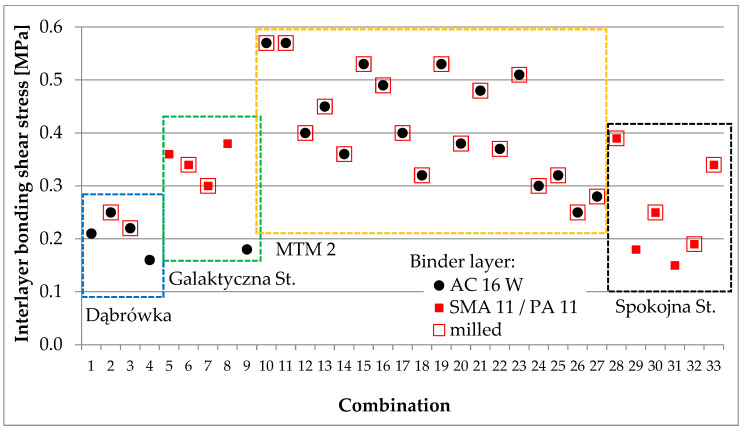
Summary of interlayer bonding shear stress results for samples from test sections.

Core samples were taken from each test section for laboratory testing. Depending on the purpose, cores with a diameter of 100 or 150 mm were taken. Void content, shear strength, and interlayer bonding were assessed according to the methodology described in Section 3.3. A summary of the test results for samples cored out from tests sections are presented in Figure 13, Figure 14 and Figure 15.

Regardless of the test section, the content of voids in the compacted poroelastic layer ranged from about 15% to about 30%. In the case of shear strength and interlayer bonding, the best results were obtained for the mixtures placed in the asphalt plant MTM. In the case of road sections, for which mixture transport time was about 2 h, the mixtures were characterised by lower internal cohesion and worse bonding. 

## 4. Characteristics of Pavements on Test Sections 

Construction of short test sections was carried out along with the progress of laboratory work on determining the composition of the poroelastic mixture in terms of wearing course durability, adhesion with the underlying layer, acoustic properties, and skid resistance. Test results of sound absorption coefficient, drainability, macrotexture, and friction coefficient, along with their analysis on short test sections built in 2018–2020, were discussed in detail in the works [25,28]. 

In September 2020, a long test section was built on Spokojna St. in Kartoszyno. Characteristics of its surface are presented in Section 3.7. Tests of the sound absorption coefficient, drainability, friction coefficient, macrotexture, and longitudinal and transverse evenness of the poroelastic pavements were carried out on full-size test sections during four measurement sessions (I—24 September 2020; II—26 October 2020; III—17 November 2020; and IV—17 March 2021), using stationary and mobile devices. 

Tests with mobile devices: road surface profiler (RSP) and skid resistance tester v.3 (SRT-3) performed in 2020 confirmed good technical condition of the new surfaces in terms of evenness and skid resistance. There were no significant differences between the poroelastic pavements and the reference SMA11 pavement. However, considerable distress of the poroelastic pavements occurred after the winter period of 2020/2021 and the results of measurements with mobile devices were not included in further analyses. 

Three measurement sessions with stationary devices were carried out in September, October, and November 2020. Before the fourth measurement session in March 2021, a detailed visual assessment of the technical condition of the pavement was performed. The tests were conducted in locations free of damage that would interfere with their adequate performance. 

The evaluation of the macrotexture was made using the circular texture meter (CTM), a laser device, in accordance with [29]. Measurements of the friction coefficient were made on the same surface using the dynamic friction tester (DFT) device in accordance with [30]. BPN values were determined with the British pendulum tester in accordance with [31]. 

Sound absorption coefficient values were determined using the Spectronics ACUPAVE System, which determines the sound absorption coefficient in accordance with [32]. The measurements were conducted after placement of the ACUPAVE on the pavement, calibration of the system, and proper placement of the microphones. The system calculated the sound absorption coefficient in 1/3 octave bands in the frequency range from 315 Hz to 1600 Hz. Pavement drainability was tested using the field method in accordance with [33]. 

**Figure 16:**
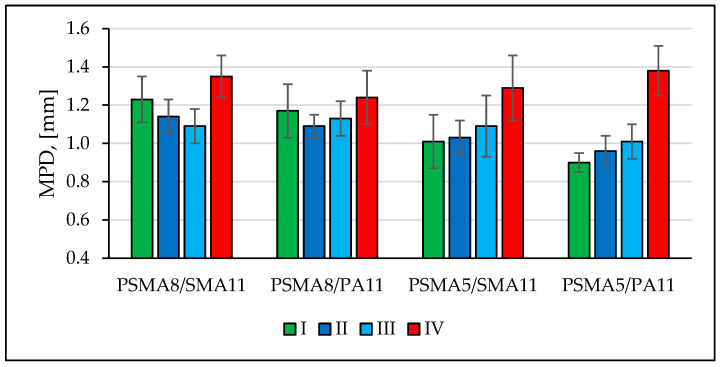
Results of macrotexture measurements on long test section in Kartoszyno.

Figure 16 shows the average macrotexture values (MPD—mean profile depth) determined during four measurement sessions. It was observed that the poroelastic mixture layers on the test sections were properly constructed from the point of view of macrotexture. No significant changes in macrotexture were noted on the wearing course in the first months of service. After the winter period of 2020/2021, due to extensive damage to the surface, it was impossible to take measurements along the entire length of the section. The results determined in March 2021 on undamaged parts of the test sections are significantly different from the previously determined values. 

**Figure 17:**
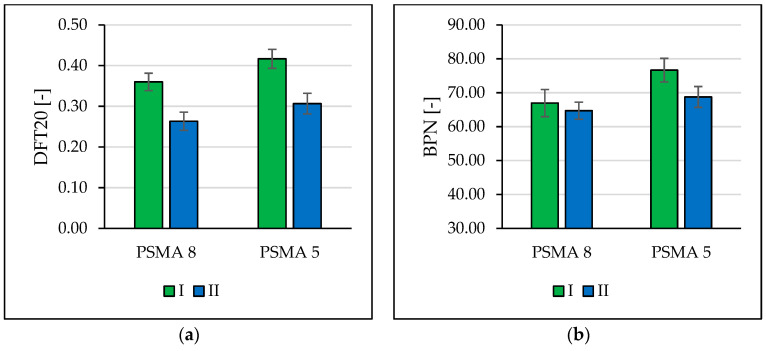
Comparison of the average values of DFT20 (**a**) and BPN (**b**) recorded in two measurement sessions.

**Figure 18:**
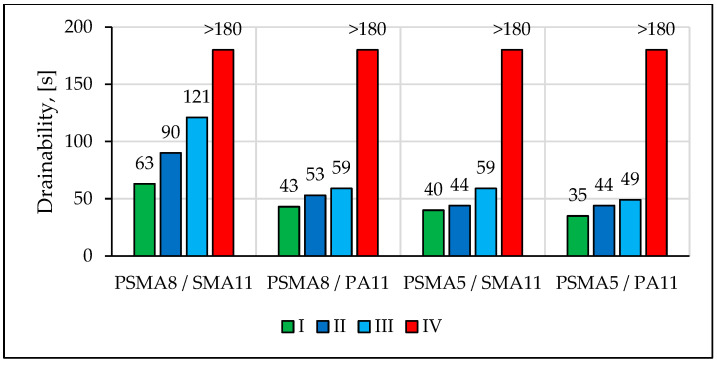
Evaluation of drainability of poroelastic pavements on test sections in four measurement sessions.

Figure 17 shows the determined mean values of DFT20 and BPN. A month after the section was opened to car traffic, a decrease in the coefficients of friction was recorded. Due to the observed damage to the pavement at some measurement points, the results of DFT20 and BPN tests in two subsequent measurement sessions were not presented. The drainage properties of the pavement deteriorated within the period of service (Figure 18). On the PSMA5 pavement, there was practically no effect of the binder course made of the PA11 mixture on its drainability compared to the binder course made of the SMA11 mixture. The influence of the type of the binder course on drainability was noted in the case of the wearing course made of the PSMA8 mixture. After the winter period, there was a significant deterioration in the drainability of the pavements on all test sections. 

**Figure 19:**
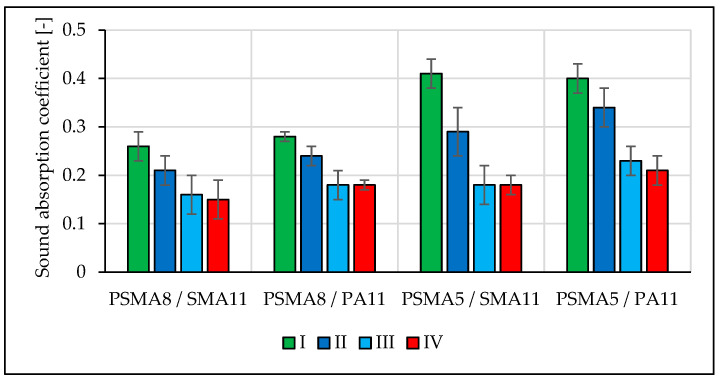
Changes in sound absorption coefficient during pavement service on test sections.

The results of the sound absorption coefficient measurements on four test sections in four measurement sessions are shown in Figure 19. 

**Figure 20:**
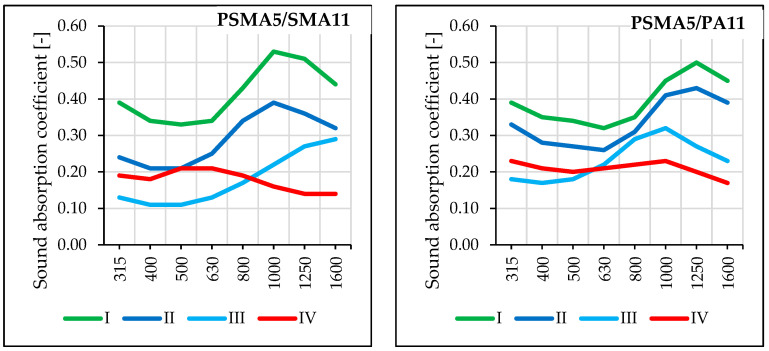
Comparison of sound absorption coefficient in the range of eight frequencies in four measurement periods on PSMA5/SMA11 and PSMA5/PA11 pavements.

The average values of sound absorption coefficient show that it decreased in time during road service. Compared to the value measured before road use, the deterioration of acoustic properties was significant. The wearing course made of PSMA5 was found to display better sound absorption capacity. Significance of the influence of the binder course type on sound absorption coefficient was not confirmed. This observation is based on the values of sound absorption coefficient for the PSMA5 pavement in eight frequency bands (Figure 20).

Based on the results of tests on short and long test sections, it was observed that the solutions adopted for the PSMA8 and PSMA5 poroelastic layers were correct. Unfavourable changes in the pavement macrotexture were related to the destruction of the structure of the wearing course consisting in the loss of rubber granulate grains in the first stage of use and the subsequent tearing out of mineral aggregate grains. The PSMA5 pavement was characterised by better water permeability compared to PSMA8. During the first months of road operation, no significant decrease in drainability was noted in the test sections. On the PSMA8 pavement, slightly better drainability was noted in the case of the PA11 porous asphalt binder course. After the winter period, drainability of the pavement in all sections decreased significantly. The reason was the destruction of the upper layer, and probably also improper winter maintenance of the road. Sound absorption coefficient decreased significantly with time. The reason was the systematic contamination of the surface by vehicles entering from the surrounding area. The influence of the type of binder layer on sound absorption coefficient was not confirmed. The results of the surface characteristics, drainability, and sound absorption coefficient of the poroelastic layers confirm that while the requirements in terms of evenness, skid resistance, and acoustic properties were fulfilled immediately after pavement construction, maintaining the stability of the structure of the poroelastic layer during the service period is still a problem. 

## 5. Noise Properties of Poroelastic Surfaces

The defined main goals of the SEPOR project included improvement of the poroelastic road surface durability through optimisation of mixture composition, improvement of the production process, and increased interlayer bonding strength. However, in the efforts to achieve such objectives, it is also crucial to maintain (or even better, to improve) the most important feature of this type of pavement, which is its remarkable traffic noise reduction capacity. The PERSUADE project [8] proved that this kind of surface has an extraordinary potential in traffic noise reduction: up to 12 dB in relation to conventional road pavements. It was assumed within the SEPOR project that the tyre/road noise reduction should be no less than 10 dB compared to the reference road surface SMA11. Noise properties of the designed poroelastic pavement were tested at all stages of the project: on small-scale test sections (width of 1.5–2.5 m and length of 10–30 m, laid on a closed small yard of an asphalt plant) of different variants of asphalt–rubber–mineral mixtures developed through theoretical considerations, on a laboratory drum facility covered with selected poroelastic road surface, on short test sections (width of 3–5 m and length of 12.5–20 m, paved on local roads with very low traffic), and on full-scale test sections (one road lane with a width of 3 m and minimum length of 100 m) of the best, most promising mixture compositions, and interface layers selected to be tested in real road traffic conditions.

### 5.1. CPX Test Trailer Method

**Figure 21:**
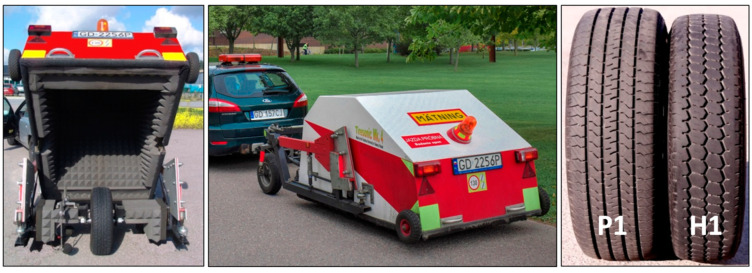
CPX test trailer Tiresonic Mk4 and the two ISO reference test tyres used: P1 and H1.

Tyre/road noise measurements were performed according to the CPX method [34], using a specialised test trailer, Tiresonic Mk 4 (Figure 21, left and middle) [35], equipped with two standard reference tyres (Figure 21, right) specified in the ISO 11819-3:2017 standard [36]: one representing the noise from passenger vehicles (P225/60R16 Uniroyal Tigerpaw “Standard Reference Test Tyre”–designated P1) and one representing the noise from heavy vehicles (195R14C Avon Supervan AV4–designated H1). Two microphones are located in close proximity of the tyre/pavement contact patch. The result of the measurements was presented as the CPX index (CPXI), which was calculated as the arithmetic mean of sound pressure levels measured for the two reference tyres.

### 5.2. CPB and SPB Methods

Tyre/road noise level tests were carried out using the controlled pass-by method (CPB) and the statistical pass-by method (SPB), according to ISO 11819-1:1997. The CPB method of controlled vehicle passage consists in measuring the maximum sound level LA_max_ from single test vehicles with known characteristics (it is recommended to use at least four vehicles with different technical characteristics) at different speeds (in steps of 10 km/h). The SPB method of statistical vehicle passage consists of measuring the maximum sound level LA_max_ from passing passenger vehicles, trucks, and multi-unit trucks selected individually from the traffic stream, with simultaneous measurement of their speed. In the two methods, the microphone was located at a constant distance of 7.5 m from the axis of the track of passing vehicles and at a height of 1.2 m above the road surface. Due to the low share of trucks in traffic and their very diverse technical conditions, the maximum sound level from the passage of this group of vehicles was not tested. 

The research program included:

- measurements using the CPB method at various stages of the service period of test sections with poroelastic wearing course and SMA11 reference wearing course,
- and rolling noise measurements using the SPB method on poroelastic wearing courses and on a new AC11 asphalt concrete wearing course.

During preliminary tests using one passenger vehicle, it was observed that in the case of the PSMA5 pavement, the type of binder course had no significant impact on the noise level. The section with the PSMA8 wearing course and the PA11 porous asphalt binder course had a slightly lower noise level compared to the SMA11 binder course (by 0.9 dB at the speed of 50 km/h and by 0.4 dB at the speed of 80 km/h). Therefore, taking into account the values of the sound absorption coefficient, a decision was made to perform further noise level tests using the CPB and SPB methods on sections with the SMA11 binder course. Maximum sound level measurements using the CPB method were carried out on four test vehicles: the Volkswagen Golf IV (tyre: 195/65 R15), Renault Scenic (tyre: 185/65 R15), Citroen C4 (tyre: 205/60 R16), and Opel Vectra (winter tyre: 215/55 R16). 

**Figure 22:**
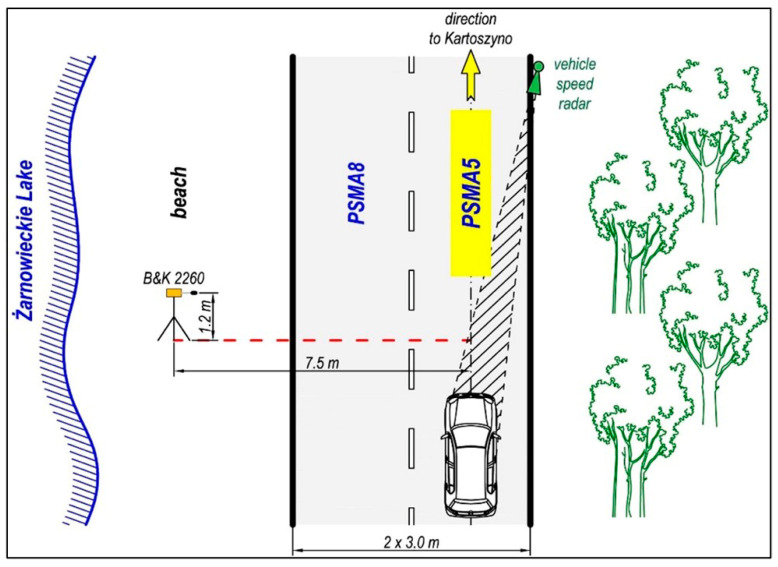
Scheme of the maximum sound level tests according to the CPB and SPB methods.

Figure 22 shows a scheme of the maximum sound level tests according to the CPB and SPB methods.

### 5.3. Noise Measurements on Short Test Sections with CPX Test Trailer

First pilot small-scale test sections of five different poroelastic mixture types were paved during technological trials described in Section 3.6 in the autumn of 2018 in three small lines (seven test sectors in total) directly at the asphalt plant yard and pre-tested for noise and rolling resistance. It should be mentioned that the paved poroelastic wearing courses were not bonded in any way to the underlying layer, which was an old dense road surface in this case. The location of the test section on a small closed yard allowed measurements at the speed of 30 km/h only. The obtained results are presented in Table 6 as noise reduction compared to the most common conventional pavement SMA11.

Unfortunately, the values of noise reduction were not fully satisfactory, but one should remember that they were obtained in the early first trials in the SEPOR project and the main goal at that stage was to verify the possibility of producing mineral–rubber–asphalt mixtures in a standard asphalt plant and the possibility of paving the produced mixture using conventional equipment for laying of typical mineral–asphalt mixtures. Noise properties were not of the greatest interest in that case. A more detailed description of that trial is presented in [37].

The second trial on several small-scale test sectors was performed in the summer of 2020. Sixteen test sectors of five poroelastic mixtures (differing in aggregate size, grading of crumb rubber aggregate, highly modified bitumen used, air voids content, binder amount, and addition of hydrated lime) were built at the same location: the asphalt plant yard. Test sectors of the same asphalt–rubber–mineral mixture varied with types of asphalt paver used, levels of initial compaction by paver screed, and/or levels of final compaction. Noise tests had the same limitations as during the first trial (test speed of 30 km/h only and impossibility of testing at first two sectors). Noise reduction in comparison to SMA11 pavement for all the investigated test sectors is shown in Table 7.

Noise reduction obtained for poroelastic mixtures during the second trial of small-scale test sections (up to 8.6 dB) was much more promising than in the first trial. The average value for all tested sections was 7.5 dB, the worst section showed a reduction of 6.0 dB only. In summary, the noise measurement results look promising, as the small-scale test sections were tested in difficult conditions and at low speed only.

### 5.4. Noise Measurements on the First Two Long Test Sections with CPX Test Trailer

The first long test section of poroelastic pavement (50-m-long) consisting of four sectors was built on the grounds of the “Gryf” Military Technology Museum in Dąbrówka in the summer of 2019. Road traffic at this location was very small, estimated to be about 150 passenger cars and 2–3 trucks and buses per day, moving at very low speeds.

The main purpose of this trial was to optimise bonding between the poroelastic wearing course and the underlying base layer. Only one type of mineral–rubber–asphalt mixture was used in this location. The designed four sectors, each of the length of 12.5 m, differed only with the interlayer connection type, which should not influence the measured tyre/road noise at all. This was proved by the preformed measurements; however, the entire test section was not as homogeneous as it was expected. The first part of this test section, 18-m-long, was very inhomogeneous, while the remaining 32 m was almost perfectly homogeneous. Thus, two separate sectors were established based on homogeneity and not on interlayer connection type. They were designated “SEPOR-D-PSMA5 W4 s1” and “SEPOR-D-PSMA5 W4 s2”. Homogeneity is a very important issue in the case of low-noise wearing courses [38].

Noise measurements were performed twice at this test section: five days after construction of the section and two and a half months later. The test speeds were 30 and 50 km/h. Test results are presented in Table 8 as the mean values of noise reduction (in comparison to the reference SMA11 wearing course).

The maximum noise reduction for the homogeneous sector measured during the first test was 10 dB. The assumed goal of the SEPOR project, in terms of noise properties of poroelastic pavement, was achieved. Unfortunately, after over two months of service, the noise reduction decreased by 1.2 dB. When the second measurement was performed, the poroelastic pavement was already distressed and significant ravelling occurred.

**Figure 23:**
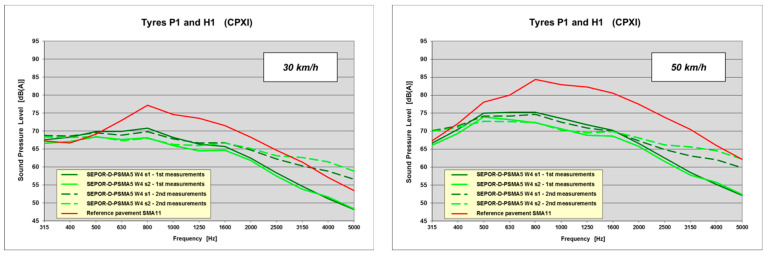
Frequency spectra of poroelastic wearing course placed at the Dąbrówka long test section.

Analysing the frequency spectra (see Figure 23), one can observe that noise reduction was obtained primarily in the medium frequency range (630–1250 Hz), mostly due to elasticity of poroelastic pavement, and in the high frequency range (1600–5000 Hz), mostly due to its porosity. During the second measurements, porosity of the pavement was significantly reduced due to contamination of the wearing course, which resulted in much higher SPL values in the high frequency range. 

Three months later, the second long test section of the total length of 160 m was built in the Galaktyczna street in Gdańsk. The measured daily traffic for this road was about 1900 vehicles, and the traffic composition was 88.4% of passenger vehicles, 6.0% of delivery vans, 4.6% of heavy trucks and buses, and 1.0% of motorcycles.

Two different types of mineral–rubber–asphalt mixtures were implemented, differing in binder content (11% or 15%). Other differences were not important from the point of view of noise properties testing: two variants of underlying pavement type and two variants of interlayer bonding (with or without glass grid). Thus, based on binder content, two sectors were evaluated separately and designated “SEPOR-G-PSMA5 W4 s1” and “SEPOR-G-PSMA5 W4 s2” (each 80-m-long). 

Noise measurements were performed twice, several days apart. First measurements were conducted the day after paving (the surface had not been subjected to road traffic yet), and the second measurements were performed 12 days later. The tests were performed at three speeds: 50, 80, and 110 km/h. The obtained results, noise reduction in comparison to the reference SMA11 pavement, are presented in Table 9.

**Figure 24:**
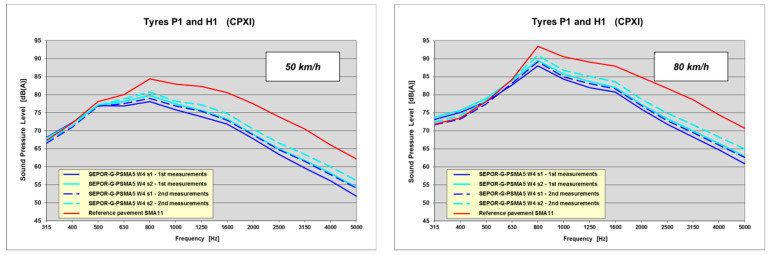
Frequency spectra of poroelastic wearing course placed at the Galaktyczna long test section.

This test section did not prove successful in terms of noise properties of the poroelastic mixtures used. The obtained maximum noise reduction was only 5.7 dB (the average was 4.3 dB). It was far from the assumed goal of 10 dB. The second measurement showed a decrease in noise reduction by about 1.0 dB. When the acquired frequency spectra were analysed (see Figure 24), it was noted that noise reduction in the medium frequency range, corresponding to elasticity of the pavement, was not as high as expected, which influenced the final results. 

### 5.5. Noise Measurements on the Third Long Test Section

#### 5.5.1. CPX Test Method

The experience gained from the aforementioned small-scale trials was used to produce the final poroelastic pavement variants to be used on the third and final long test section. Such a section was constructed in the summer of 2020 in Kartoszyno. Four sectors of poroelastic wearing courses were built on a road with average annual daily traffic of 2500 vehicles, usually travelling at speeds in the range of 50–70 km/h. Two types of mineral–rubber–asphalt mixtures (“SEPOR-K-PSMA8 W6” and “SEPOR-K-PSMA5 W2”), differing in maximum aggregate size, added rubber aggregate fractions, and air voids content, were laid on two different base layers: SMA11 (designated with the suffix “DB” for “dense base-layer”) and PA11 (designated with the suffix “PB” for “porous base-layer”). The lengths of the test sectors laid on SMA11 and PA11 were 200 m and 50 m, respectively. 

**Figure 25:**
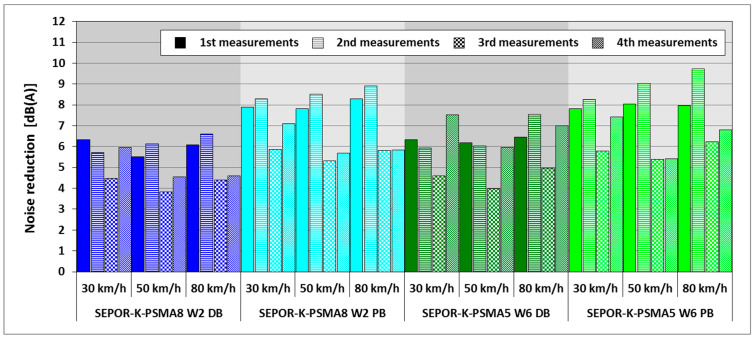
Noise reduction of poroelastic mixtures on the Kartoszyno long test section.

Due to unexpected problems with the durability of laid pavements, the wearing courses were repaved with exactly the same mixtures shortly after construction. Noise measurements were performed four times in total: 1 week after the pavement was laid for the first time, then 2 weeks later, and 2 days after repaving, and then 5 weeks later. The test speeds were 30, 50, and 80 km/h. Results obtained during all the tests are shown in Table 10 and Figure 25 as noise reduction in comparison to the SMA11 pavement.

The maximum noise reduction obtained on the final Kartoszyno long test section was 9.7 dB, which is a very satisfactory result. Unfortunately, the average value of noise reduction was much lower. Depending on the test sector, it ranged from 5.4 to 7.3 dB. Higher noise reduction (by 1.3–1.8 dB) was obtained for poroelastic pavement placed on the porous base layer than for the pavement placed over the dense layer. Higher values were also noted for the original pavements placed in the first attempt. In general, the lowest sound values for all pavements and almost all test speeds were obtained in the second measurement. Although the mineral–rubber–asphalt mixtures should be the same after repaving, their noise reduction properties were lower by about 0.7–1.0 dB (for sectors placed on dense base layer) to 2.3 dB (for sectors placed on porous base-layer) in the second attempt. A possible explanation is that the porosity of porous asphalt in the base layer was significantly reduced during repaving.

**Figure 26:**
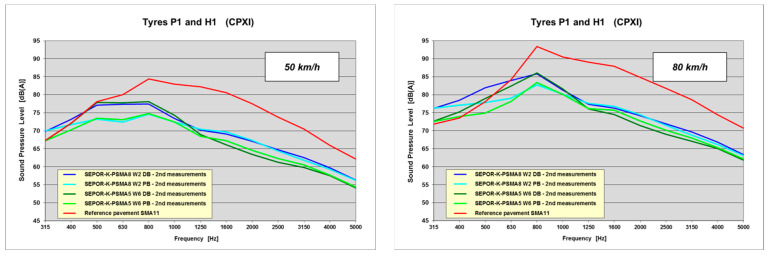
Frequency spectra of poroelastic wearing courses on the Kartoszyno long test section.

Frequency spectra obtained at 50 and 80 km/h for all the tested pavements in the second measurement, when the highest noise reduction was noted, are presented in Figure 26.

Based on the frequency spectra, it can be stated that noise reduction occurred primarily in the medium and high frequency ranges, which is typical for poroelastic wearing courses. The influence of porosity of the underlying layer was also clearly visible for the low frequency range. The average noise reduction for medium frequencies (630–1250 Hz) was over 10 dB, while the maximum was 13.5 dB. For high frequencies (1600–5000 Hz) the average reduction was also over 10 dB and the maximum equalled 14.0 dB. 

In conclusion, in all the noise trials performed within the SEPOR project, two designed variants of mineral–rubber–asphalt mixtures designated “SEPOR-D-PSMA5 W4” and “SEPOR-K-PSMA5 W6 PB” fulfilled the noise requirements assumed at the beginning of the project: noise reduction of about 10 dB in comparison to the most popular conventional wearing course SMA11. Unfortunately, their durability was below expectations, they wore down in a relatively short time, significant ravelling occurred and the test sections had to be removed for safety reasons. However, the obtained results are similar or only slightly lower than the values for the PERS poroelastic pavement developed under the 7PRUE PERSUADE project [8], in which polyurethane binders were used. A historical review of over 50 years of trials of poroelastic road surfaces presented in [8,15,39,40,41,42,43] shows that these attempts were ended with sometimes impressive (up to 14 dB), satisfactory (about 10 dB), unsatisfactory (less than 6 dB) or, more often, moderate (7–9 dB) effects regarding the obtained noise reduction. The maximum reduction of about 14 dB obtained within the SEPOR project qualifies this trial to be satisfactory in terms of its noise abatement properties.

#### 5.5.2. CPB and SPB Methods

**Figure 27:**
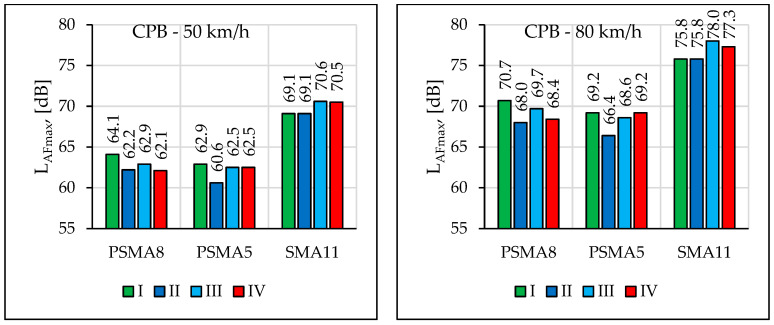
Comparison of pavement noise in four measurement periods.

Figure 27 presents the noise level values from a statistical passage of a passenger vehicle at speeds of 50 km/h and 80 km/h, determined during four measurement sessions on the PSMA8 and PSMA5 pavements and on the reference pavement SMA11. Table 11 summarises the differences in noise levels between the SMA11 and PSMA8/PSMA5 pavements.

**Figure 28:**
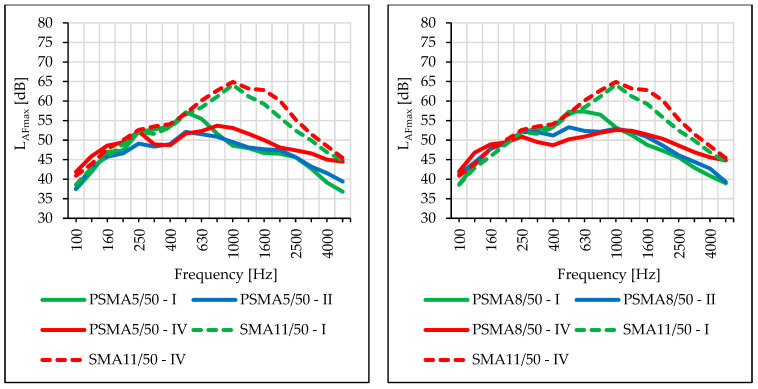
Comparison of sound spectra from a statistical passenger vehicle depending on the period of road use (PSMA5 and PSMA8).

The impact of road service life and the differences in noise levels between pavements are visible in the sound spectra (Figure 28). Increased noise levels in the frequency range of 250–800 Hz on the PSMA5 and PSMA8 pavement were noted during the first measurement session. The fact was caused by technological unevenness that occurred after the construction of the poroelastic layer. It was noted that the acoustic properties of the pavement deteriorated with its service time, especially in the case of the PSMA5 pavement in the frequency range of 800–1250 Hz. This fact was caused by the clogging of free spaces and structural damage to the pavement. Similar influence of service time and clogging was noted in the work [44].

During the fourth measurement session, temperature and pavement moisture were slightly lower. This affected the measurement results, which is clearly visible in the case of the SMA11 pavement in the frequency range of 1250–5000 Hz. 

**Figure 29:**
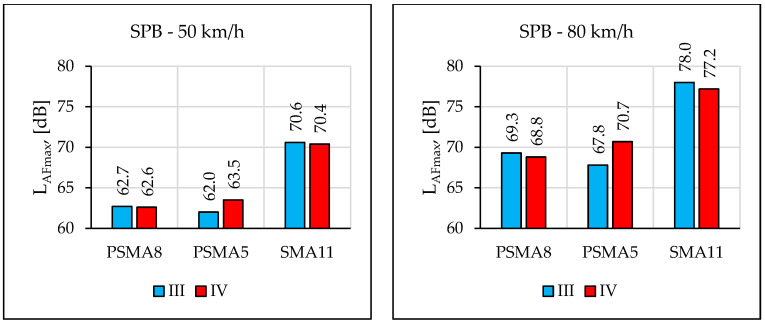
Comparison of the maximum sound levels according to the SPB method obtained in two measurement sessions.

**Figure 30:**
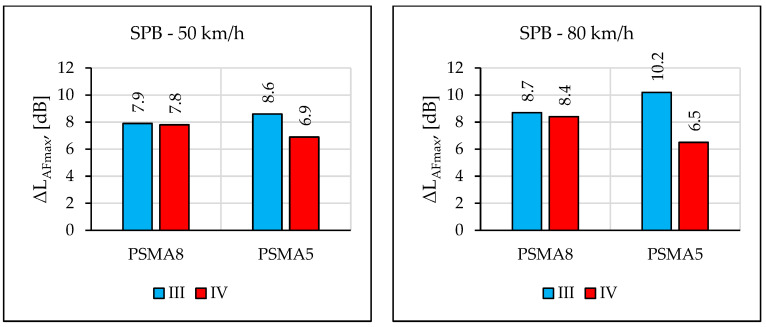
Differences between the maximum sound levels according to the SPB method obtained for the SMA11 pavement and the given poroelastic pavement.

Measurements of the maximum sound level using the SPB method were made during two measurement sessions (III and IV) on the PSMA8, PSMA5, and SMA11 pavements. Figure 29 shows the determined values of the maximum sound level and Figure 30 shows the differences between the values determined on the SMA11 pavement and the values obtained on the poroelastic pavements. 

The results of noise level measurements according to the CPB and SPB methods confirmed favourable acoustic properties of the poroelastic pavements—also after the period of winter service. However, this statement applies only to some of the test sectors, where poroelastic wearing course was not damaged. The rolling noise level tests according to the SPB method, carried out in March 2021, showed significantly worse acoustic properties of the PSMA5 pavement compared to the PSMA8. During the measurements on 17 November 2020, the difference in noise levels of the PSMA5 and SMA11 pavements at the speed of 80 km/h was 10.2 dB, and on 17 March 2021 it decreased to 6.5 dB. Such a change was not observed on the PSMA8 pavement. This was due both to clogging of free spaces in the PSMA5 pavement and its greater damage. Similar events were also observed in other research works [44,45,46]; concerning low-noise porous pavements, the acoustic properties of the pavements decreased significantly over the service period.

The results of the maximum sound level measurements using the CPB and SPB methods on long test sections on the PSMA8 and PSMA5 pavements and on the reference pavement SMA11 justify the conclusion that the designed poroelastic mixtures and the constructed PSMA pavements, if maintained in good technical condition, can reduce rolling noise of a statistical passenger vehicle by about 10 dB in comparison to the SMA11 pavement. 

Similar results were obtained in [47]. In the case of the speed of 50 km/h, the average value of the maximum sound level on the PERS pavement was approximately 3 dB(A) higher than on the PSMA5 and PSMA8 pavements. However, at the speed of 80 km/h, the difference decreased to about 2 dB. Compared to the reference surface (dense asphalt concrete–DAC), the PERS surface proved quieter by about 7–8 dB. Greater tyre/road noise reductions (of 8–10 dB) were obtained on PSMA pavements. The situation is similar in Japan, where noise reduction on a poroelastic road surface was about 10 dB.

The pavement with PSMA5 wearing course was a better solution in terms of noise reduction than the pavement with PSMA8. The average difference between the maximum sound levels from the passage of a passenger vehicle on new pavements was approximately 1.5 dB. However, over the period of service, acoustic properties of the PSMA5 surface deteriorated faster. No significant influence of the PA11 porous asphalt binder course on the maximum sound level was confirmed for a passenger vehicle travelling at 50 km/h and 80 km/h (in comparison to the SMA11 binder course). Pavements with a poroelastic layer proved noisier (by about 2 dB) immediately after placement than after a month of use. This was probably caused by single grains of aggregate and rubber granulate remaining on their surface immediately after pavement construction. 

In order to summarise the results of the tests using the CPB and SPB methods, the values of the differences between the maximum sound levels from the passage of a passenger vehicle on the PSMA8/PSMA5 pavements and on the SMA11 reference pavement were analysed (based on the results obtained during the third measurement session, when the surface was in acceptable condition). In the case of the PSMA8 pavement, the differences were 7.7 dB (50 km/h) and 8.3 dB (80 km/h) according to the CPB method and 7.9 dB (50 km/h) and 8.7 dB (80 km/h) according to the SPB method. In the case of the PSMA5 pavement, the differences were 8.1 dB (50 km/h) and 9.4 dB (80 km/h) according to the CPB method, and 8.6 dB (50 km/h) and 10.2 dB (80 km/h) according to the SPB method. The results indicate that in terms of acoustic properties, a new PSMA5 poroelastic wearing course is a better solution than PSMA8. However, the wearing course made of the PSMA5 mixture loses its favorable acoustic parameters quicker.

## 6. Rolling Resistance

Rolling resistance belongs to the most important operational parameters that characterise the interaction between the tyre and the road surface. It depends largely on tyre structure (tyre geometry, tread, carcass and belt materials, and tread pattern) as well as on the texture and stiffness of the road surface. Paradoxically, the changes that take place in the car fleet and vehicle operating conditions cause an increase in the share of energy loss resulting from rolling resistance in the overall energy consumption of vehicles. Energy losses related to rolling resistance depend on the speed of the vehicle to a very limited degree, so the global trend of lowering the average traffic speed does not have a significant effect on these losses, as opposed to aerodynamic losses, which are lower. On the other hand, in hybrid and electric cars, which are increasingly popular, the losses associated with acceleration and braking are reduced, as the energy is largely recovered and stored in batteries for further use. Unfortunately, the trade-off lies in the associated increase in the weight of such vehicles, translating into a proportional increase in losses related to rolling resistance.

Regulations regarding the approval of tyres for commercial use require that the tyres be marked with labels providing information about the quality of the product in terms of energy losses (i.e., rolling resistance), noise, and traction. Unfortunately, the conditions for performance of measurements defined in the labelling procedures pursuant to the ECE Regulation No. R117 are highly unrepresentative and the obtained results do not correlate well with the actual performance of tyres. For example, rolling resistance is tested on a smooth steel drum, i.e., a surface that is never encountered in road conditions.

Recently, tyre manufacturers made great progress in reducing the rolling resistance of tyres, which is often characterised by the coefficient of rolling resistance (CRR), which is the ratio of the rolling resistance force to the tyre vertical load. While 20 years ago the CRR value of 0.01 (for the speed of 80 km/h and for a smooth steel drum) was considered outstanding, many tyres nowadays display CRR values close to 0.007 under the same conditions. It is estimated that a 30% reduction in rolling resistance corresponds to an 8–10% reduction in the overall energy consumption of passenger cars.

Optimisation of all experimental poroelastic pavements is carried out based on the tyre/road noise criterion, because these surfaces display the greatest potential in this respect; however, it is obvious that the rolling resistance, while less important for this pavement, must also be at an acceptable level. An obstacle to determining the rolling resistance of poroelastic surfaces in relation to conventional surfaces is the lack of standard methods for rolling resistance measurements that would take into account the impact of the road pavement. For this reason, the researchers at the Gdańsk University of Technology had to use own methods that were not standard methods defined by the formal ISO or ECE standards and regulations.

**Figure 31:**
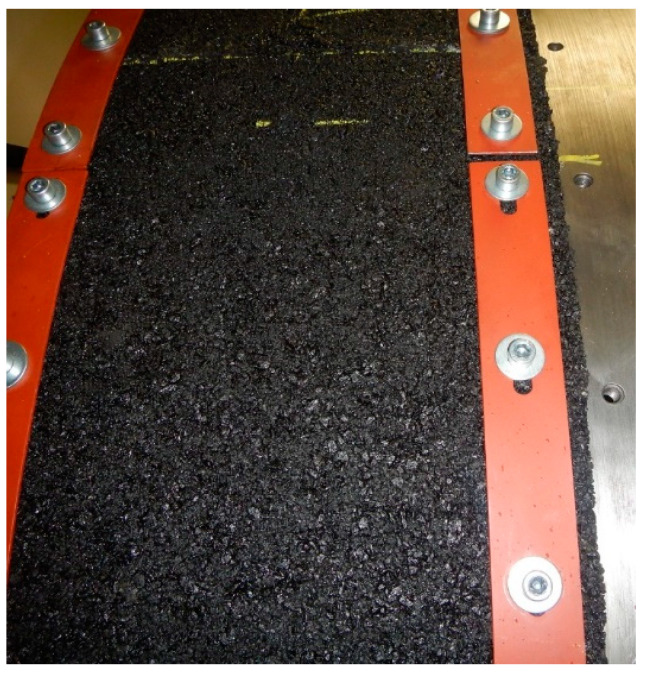
SEPOR plates mounted on the drum.

**Figure 32:**
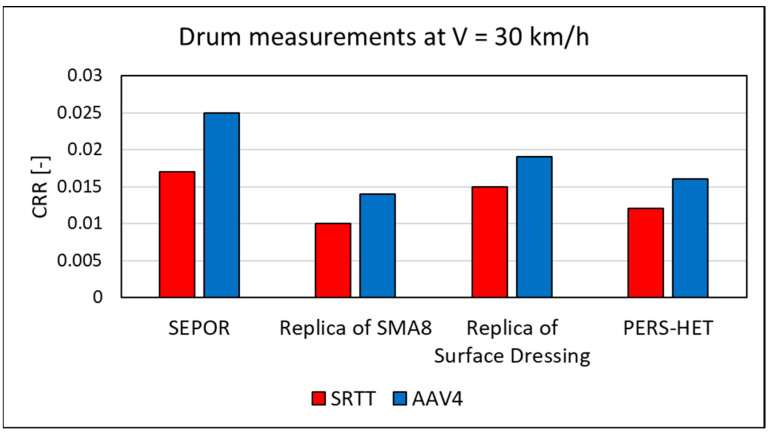
Coefficients of rolling resistance measured at 30 km/h on the roadwheel device at 25 °C.

Therefore, a method of covering the steel surface of the drum of a running machine adapted to measuring the rolling resistance with SEPOR-type porous pavement patches was developed—see Figure 31. Due to the risk of detachment of the SEPOR surfacing from the drum, it was necessary to limit the test speed to 30 km/h. The obtained results are presented in Figure 32, which compares the rolling resistance coefficients for two reference tyres selected according to ISO/TS 11819-3 (SRTT is Standard Reference Test Tyre and AAV4 is test tyre with characteristics similar to truck tyres) tested at the temperature of 25 °C. Regulated inflation pressure of 210 kPa and vertical load of 4000 N were used for all the rolling resistance tests. The coefficients were obtained on the SEPOR surface and on the replicas of stone mastic asphalt (SMA8), surface dressing (APS4), and a poroelastic surface based on polyurethane binder (PERS-HET). The results indicate that the SEPOR pavement shows the highest rolling resistance among the tested surfaces, and the increase in relation to surface dressing is within the range of 10–30%. The authors, however, consider the drum method to be unsatisfactory for testing of the SEPOR pavement, as at the speed of 80 km/h, any given point of the surface is in contact with the tyre every 0.3 s, which increases the temperature of the material considerably. Such conditions would never occur on a real road.

**Figure 33:**
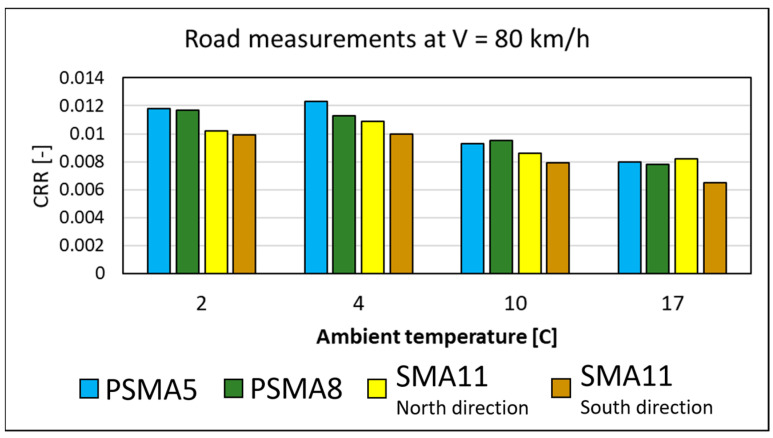
Coefficients of rolling resistance measured on the road at 80 km/h at different ambient temperatures.

Due to the fact that laboratory tests using the bonded SEPOR material plates could not be carried out at higher speeds, the main measurements were performed in field conditions using the R2 Mk.2 experimental trailer. The measurements were taken on the Kartoszyno final long test section throughout its existence at temperatures ranging from 2 °C to 17 °C. Figure 33 shows the CRR values obtained at 80 km/h for a typical high-quality passenger car tyre (Goodyear Efficient Grip Performance, 225/60R16, energy label “A”). As shown in the figure, the rolling resistance on the SEPOR surfaces PSMA5 and PSMA8 is only slightly higher than the resistance on the reference wearing course SMA11 [48,49].

In summary, although the tested poroelastic pavements displayed worse rolling resistance properties than the reference pavements, the difference was not critical, and it should be considered that the poroelastic pavements show acceptable values of rolling resistance at the present stage of development.

## 7. Fire Hazard

One of the primary features of poroelastic pavements is their very positive effect on the suppression of spilled fuel fires. These features were noticed already during the implementation of the 7PRUE PERSUADE project in relation to poroelastic surfaces produced with polyurethane binder. As the pavements developed under the SEPOR project were based on bituminous binder, it was necessary to verify whether these pavements also exhibited advantageous fire suppression properties.

Fires of engine fuel spills under the chassis of a passenger car were investigated. In order to carry out the tests, four Opel Corsa passenger cars (withdrawn from traffic) were purchased. Four test pads were prepared and covered with the following pavements:

- porous asphalt PA11,
- SMA 8;
- poroelastic surface developed in the PERSUADE project, based on polyurethane binder–PERS-HET;
- and poroelastic pavement developed in the SEPOR project, based on highly modified asphalt binder–SEPOR.

**Figure 34:**
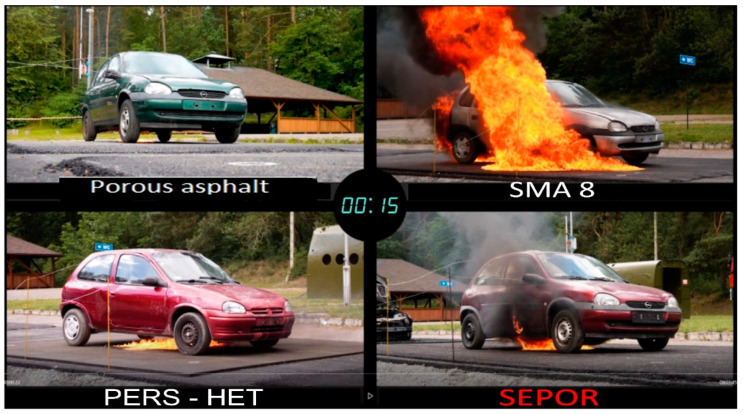
Fire development 15 s after ignition.

**Figure 35:**
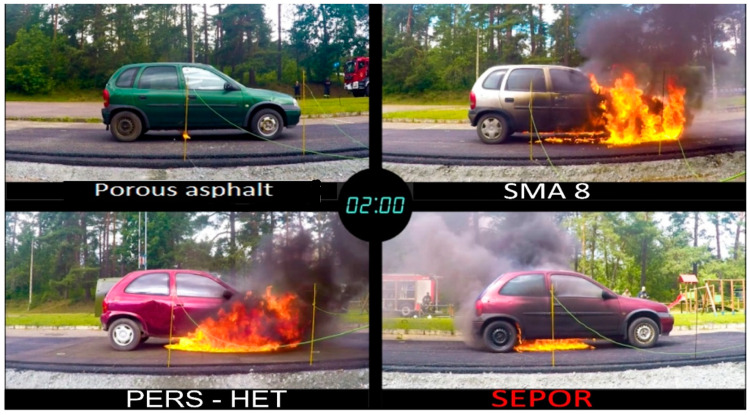
Fire development 120 s after ignition.

One car was placed on each pad; 15 L of gasoline fuel were poured under each car and set on fire. The course of the fires was monitored using cameras and gas sensors. Figure 34 shows development of the fire after 15 s from ignition for each of the tested pavements. The situation after 120 s is shown in Figure 35. It is clearly visible that the fire intensity is significantly reduced on porous surfaces in relation to the dense SMA8 wearing course. It is clear that the SEPOR pavement contributes to suppression of fire, but the damping effect is not as pronounced as in the case of a conventional porous asphalt PA11. Observations of the fuel spills showed that the SEPOR pavement did not absorb fuel as effectively as the typical porous asphalt. This fact could be associated with the use of anti-adhesive agents during compaction, which were not washed away by rain. Tests of air quality at the distance of 5 m from the fire did not reveal the presence of hydrogen cyanide, ammonia, carbon monoxide, or chlorine.

The experiment confirmed a very positive impact of porous surfaces on safety in the event of fuel spill fires, which undoubtedly predisposes such surfaces to usage in tunnels or at fuel stations.

## 8. Conclusions and Further Research

The SEPOR project was intended for development of an innovative road pavement characterised by low noise, good durability, moderate rolling resistance, and the capacity to limit the spread of spilled fuel fires.

It was assumed that research works would concern poroelastic wearing courses, because such pavements, according to the literature review and earlier experience, can significantly reduce tyre/road noise (by 10–12 dB) in comparison with reference typical wearing courses, e.g., stone mastic asphalt SMA11. Previous research efforts concerning poroelastic pavements, which were almost exclusively based on polyurethane binder (PERS), indicated that while the reduction in tyre/road noise by 10–12 dB was possible to achieve, the durability of such mixtures was insufficient. The main distress mechanism involved loss of internal cohesion, fast ravelling, and debonding from the layer underneath. Despite numerous attempts to improve bonding between the PERS mixture and the base layer, interlayer bonding was insufficient, and in many cases, due to specific stress distribution in the contact zone, even cracking of the base course occurred.

Thus, during the SEPOR project, the decision was made to replace the previously used polyurethane resin binder with highly polymer-modified bitumen, which was available on the road materials market for some time. After numerous laboratory and full-scale trials, a new type of mineral–rubber–asphalt mixture was developed. This mixture was used to construct the experimental test section located in Kartoszyno. The section was constructed in autumn 2020 and it remained in service over the winter period until April 2021, when it proved necessary to replace the poroelastic wearing course with traditional wearing course SMA11 due to severe distress that occurred on one lane.

Tests that were carried out on this section showed that reduction in tyre/road noise was within the range of 6 to 14 dB, depending on instantaneous conditions (the most significant reduction was observed for wet pavement). Rolling resistance was similar to typical wearing courses (e.g., asphalt concrete and stone mastic asphalt). Skidding resistance was good, regardless of weather conditions (dry, rain, and snow). The issue of premature debonding of the poroelastic layer from the base layer was eliminated entirely, despite harsh winter conditions that strongly affect interlayer bonding of such porous layers with the layer underneath. Unfortunately, resistance to ravelling and internal cohesion proved insufficient. Due to a lack of funding necessary to construct another test section and the approaching project completion date, the SEPOR project was discontinued.

Comparing the outcome of the SEPOR project with the PERS poroelastic mixture, it can be stated that it was possible to reduce rolling resistance, improve skidding resistance regardless of weather conditions (on a wet or snowy pavement it was even better than in the case of typical asphalt pavement), and greatly simplify the technological process, so that it was possible to use ordinary construction equipment during production and laying of poroelastic mixtures. The unit cost of poroelastic pavement was significantly reduced and the problem of debonding of the poroelastic layer from the base layer was solved. Unfortunately, the issue of insufficient internal cohesion and resistance to ravelling could not be solved, which resulted in insufficient durability of pavement in the field.

Nevertheless, members of the research team are convinced that the experience gained during the implementation of the SEPOR project is very valuable and will serve as the basis for further development of the poroelastic mixture, ultimately leading to its commercialisation.

## Figures and Tables

**Table 1:** Summary of the development of poroelastic pavements.

Country/Year	Rubber Particles [%, m/m]	Natural Aggregate [%, m/m]	Binder [%, m/m]	Notes
Sweden 1979 [2]	granulate	-	bitumen 200–500	~10 dB of reduction
Netherlands 1986 [3]	-	8/11 mm–87% 0/2 mm–11%	bitumen 80/100 modified by rubber and mineral oil	air voids ~25%, thickness 35–40 mm ~8 dB of reduction
Japan 2004 [4,5]	granulate 1 mm or fibre–30%	Silica sand 0/5 mm ~60% Yoko 2.5–3 mm	urethane	air voids ~30–40% ~10 dB of reduction
Sweden 2005–2009 [6,7]	0/0.5 mm–3.8% 0.5/1 mm–4.7%	Filler – 6.5% 0/4 mm–3.5% 4/8 mm–81.5%	polymer-modified bitumen PmB20-7.8%	Bitumen emulsion BE50R 160/220 for the pre-treatment of rubber particles, ~6 dB of reduction
Sweden 2010 [8]	4/8 mm–60–90% filler 0–5%	sand 0/1 mm–0–10%	melamine 0–10% polyurethane 10–15%	~10 dB of reduction
Sweden 2016 [9]	1.6–2.7 mm–57%	sand 0/1 mm 29%	pre-polymerised polyurethane-14%	8–12 dB of reduction
China/Germany 2017 [10]	0.2–0.8 mm–5–8% 3.1–6.0 mm–80–90%	sand 0/2 mm– 2% or 15%	polyurethane – Elastopave 6551/102– 15%	-
Poland 2019 [11,12]	2/4 mm–15%	h. lime–2% 0/2 mm–5% 4/5 mm–70%	highly polymer-modified bitumen 45/80–80–10%	air voids ~15–20% 10–12 dB, max. 14 dB
China 2022 [13,14]	1.2–4.7 mm–25%	4.7/16 mm–75%	polyurethane–4%	air voids ~15–20%, potassium permanganate and UV irradiation for the pre-treatment of rubber particles

**Table 2:** Properties of bitumens.

		Type of Bitumen		
Property		10/40–65	45/80–80	65/105–80
Penetration at 25 °C, 0.1 mm, acc. to PN-EN 1426	Original	28	53	87
	RTFOT	20	40	68
R&B temperature, °C, acc. to PN-EN 1427	Original	73.6	78.7	90.5
	RTFOT	77.3	87.8	91.4
Performance Grade, acc. to AASHTO M 320		86−16	82−22	76−28
Fraass breaking point, °C, acc. to PN-EN 12593	Original	not tested	−20.9	−21.2
Dynamic viscosity @135 °C, Pa*s, acc. to PN-EN 13302	Original	not tested	2.20	2.03
	RTFOT	not tested	3.33	2.80
Elastic recovery @25 °C, %, acc. to PN-EN 13398	Original	not tested	95.3	95.2
	RTFOT	not tested	91.8	94.4

**Table 3:** Composition of poroelastic SEPOR mixtures.

	Material, % (m/m)								
	Coarse Aggregate 5.6/8	Coarse Aggregate 4/5.6	Coarse Aggregate 2/5.6	Fine Aggregate 0/2	Limestone Filler	Crumb Rubber 0.5/2	Crumb Rubber 1/4	Crumb Rubber 2/4	Crumb Rubber 4/7
Stage I									
PSMA 5	0	0	58	13	9	0	0	20	0
Stage II									
PSMA 8	70	0	8 ÷ 0 *	8	12	2 ÷ 10 *	0	0	0
PSMA 8	70	0	8 ÷ 0 *	8	12	0	2 ÷ 10 *	0	0
PMNU 8	72	0	8 ÷ 0 *	12	4	2 ÷ 10 *	0	0	0
PMNU 8	72	0	8 ÷ 0 *	12	4	0	2 ÷ 10 *	0	0
Stage III									
PSMA 5	0	0	74	6	10	5	5	0	0
PSMA 5	0	0	69	6	10	5	10	0	0
PSMA 8	70	0	4	4	12	3	5	0	2
PSMA 8	65	0	4	4	12	3	8	0	4
Stage IV									
PSMA 5 W3	0	0	60	10	15	5	10	0	0
PSMA 5 W4	0	0	72	6	7	5	10	0	0
PSMA 8 W3	60	0		8	15	3	10	0	4
PSMA 8 W4	68	0	4	4	9	3	8	0	4
Stage V									
PSMA 5 W7	0	70	0	5	10	0	0	15	0
PSMA 5 W7 Hyd. Lime	0	70	0	5	8 + 2	0	0	15	0
PSMA 8 W8	70	0	0	5	10	0	0	15	0
PSMA 8 W8 Hyd. Lime	70	0	0	5	8 + 2	0	0	15	0
PSMA 8 W9	70		0	5	10	0	0	0	15

*—2% of mineral aggregate 2/5 was replaced with 2% of crumb rubber. W3, W4, W7, W8, and W9—variants of poroelastic mixtures.

**Table 4:** Summary of laboratory test methods.

Property	Standard No.	Method of Compaction acc. to EN 13108-20	Test Conditions
Stiffness modulus	EN 12697-26	C.1.2, acc. to EN 12697-30, 2 × 50 blows	IT-CY (annexe C), temperature of +25 °C, horizontal deformation of 5 μs
Indirect tensile strength	EN 12697-23	C.1.2, acc. to EN 12697-30, 2 × 50 blows	Temperature of +25 °C, rate of deformation 50 mm/min
Compressive strength		C.1.2, acc. to EN 12697-30, 2 × 50 blows	Temperature of +25 °C, rate of deformation 18 mm/min
Particle loss of porous asphalt specimens (Cantabro test)	EN 12697-17	C.1.2, acc. to EN 12697-30, 2 × 50 blows	Temperature of +25 °C, 300 revolutions
Internal shear strength	EN 12697-48	C.1.2, acc. to EN 12697-30, 2 × 50 blows	Temperature of +20 °C, rate of deformation 50 mm/min
Resistance to permanent deformation	EN 12697-22	C.1.20, acc. to EN 12697-33 P98-P100	Method B, in air, temperature of 60 °C 10,000 cycles
Interlayer bonding	EN 12697-48	C.1.20, acc. to EN 12697-33 P98-P100	Temperature of +20 °C, rate of deformation 50 mm/min

**Table 5:** Summary of laboratory test results.

Type of Mixture	Bitumen Content, % m/m	Crumb Rubber Content, %m/m	Void Content, %	Stiffness, MPa	Indirect Tensile Strength, kPa	Cantabro Loss, %	Shear Strength, MPa	Proportional Rut Depth, %	Wheel Tracking Slope, m/1000 Cycles
Stage I									
PSMA 5 45/80-80	12.0	20 CR 2/4	19.9	221	216	2	0.58	n.t.	n.t.
PSMA 5 10/40-65	12.0	20 CR 2/4	19.1	234	246	n.t.	0.64	46.3	0.41
PSMA 5 10/40-65	12.0 *	20 CR 2/4	22.9	252	191	n.t.	0.49	n.t.	n.t.
PSMA 5 10/40-65	12.0 **	20 CR 2/4	22.9	219	216	n.t.	0.53	n.t.	n.t.
PSMA 5 10/40-65; 19 mm fibres	12.0	20 CR 2/4	21.3	231	257	4	0.65	43.7	0.39
PSMA 5 10/40-65; 38 mm fibres	12.0	20 CR 2/4	22.2	218	234	12	0.58	n.t.	n.t.
PSMA 5 10/40-65; 50 mm fibres	12.0	20 CR 2/4	20.8	206	237	3	0.58	n.t.	n.t.
Stage II									
PSMA 8 45/80-80	7.9	8 CR 0.5/2	3.4	510	n.t.	n.t.	1.18	6.1	0.06
PSMA 8 45/80-80	7.9	8 CR 1/4	7.0	340	n.t.	n.t.	1.11	6.7	0.06
PMNU 8 45/80-80	6.3	4 CR 0.5/2	12.4	706	n.t.	n.t.	1.10	3.8	0.04
PMNU 8 45/80-80	6.3	4 CR 1/4	14.9	415	n.t.	n.t.	1.02	6.9	0.09
Stage III									
PSMA 5 W2 45/80-80	10.0	5 CR 0.5/2; 10 CR 1/4	16.6	n.t.	n.t.	n.t.	0.74	35.4	0.51
PSMA 8 W2 45/80-80	8.0	3 CR 0.5/2; 8 CR 1/4; 4 CR 4/7	15.7	n.t.	n.t.	n.t.	0.70	27.9	0.26
Stage IV									
PSMA 5 W3 45/80-80	10.0	5 CR 0.5/2; 10 CR 1/4	12.9	n.t.	n.t.	n.t.	0.82	40.4	0.38
PSMA 5 W4 45/80-80	11.0	5 CR 0.5/2; 10 CR 1/4	14.5	n.t.	n.t.	n.t.	0.77	46.7	0.78
PSMA 8 W3 45/80-80	10.0	3 CR 0.5/2; 10 CR 1/4; 4 CR 4/7	14.9	n.t.	n.t.	n.t.	0.78	34.8	0.27
PSMA 8 W4 45/80-80	11.0	3 CR 0.5/2; 10 CR 1/4; 4 CR 4/7	15.7	n.t.	n.t.	n.t.	0.77	33.3	0.31
Stage V									
PSMA 5 W7 45/80-80	10.0	15 CR 2/4	19.8	n.t.	n.t.	n.t.	0.55	n.t.	n.t.
PSMA 5 W7 45/80-80 Hyd. Lime	10.0	15 CR 2/4	20.2	n.t.	n.t.	n.t.	0.61	n.t.	n.t.
PSMA 5 W7 45/80-80 Sasobit	10.0	15 CR 2/4	21.7	n.t.	n.t.	n.t.	0.55	n.t.	n.t.
PSMA 5 W7 65/105-80	10.0	15 CR 2/4	16.9	n.t.	n.t.	n.t.	0.49	n.t.	n.t.
PSMA 5 W7 65/105-80 Hyd. Lime	10.0	15 CR 2/4	17.5	n.t.	n.t.	n.t.	0.47	n.t.	n.t.
PSMA 8 W8 45/80-80	10.0	15 CR 2/4	14.6	n.t.	n.t.	n.t.	0.67	n.t.	n.t.
PSMA 8 W8 45/80-80 Hyd. Lime	10.0	15 CR 2/4	15.4	n.t.	n.t.	n.t.	0.72	n.t.	n.t.
PSMA 8 W8 45/80-80 Sasobit	10.0	15 CR 2/4	16.3	n.t.	n.t.	n.t.	0.64	n.t.	n.t.
PSMA 8 W9 45/80-80	10.0	15 CR 4/5, 6	18.7	n.t.	n.t.	n.t.	0.52	n.t.	n.t.

* bitumen blended with CR before mixing process, ** rubber modification of bitumen (wet method), and n.t.—not tested.

**Table 6:** Noise reduction of prototype poroelastic mixtures obtained on the first short test section.

Test Sector	Mixture No.	Noise Reduction	Comments
		CPXI 30 km/h [dB(A)]	
MTM1-1	Mix 1	5.4	
MTM1-2	Mix 2	6.2	the highest
MTM1-3	Mix 3	5.2	
MTM1-4	Mix 4	5.4	
MTM1-5	Mix 4	5.1	
MTM1-6	Mix 5	5.1	
MTM1-7	Mix 5	3.8	the lowest

**Table 7:** Noise reduction of poroelastic mixtures obtained on the second short test section.

Test Sector	Mixture No.	Paver Type	Screed Settings	Number of Roller Passes	Noise Reduction	Comments
					CPXI 30 km/h [dB(A)]	
MTM2-1	Mix 2	small size	tampers only	3	-	not tested
MTM2-2				7	-	not tested
MTM2-3			tampers and vibrating plates	3	7.9	
MTM2-4				7	7.1	
MTM2-5	Mix 1		tampers only	3	7.4	
MTM2-6				7	6.6	
MTM2-7	Mix 4	standard size	tampers only	3	8.5	
MTM2-8				7	8.3	
MTM2-9	Mix 5			3	8.6	the highest
MTM2-10				7	7.8	
MTM2-11			tampers and vibrating plates	3	8.4	
MTM2-12				7	7.3	
MTM2-13	Mix 1		tampers only	3	6.8	
MTM2-14				7	6.0	the lowest
MTM2-15	Mix 3			3	7.4	
MTM2-16				7	6.9	

**Table 8:** Noise reduction of poroelastic mixture placed at the Dąbrówka long test section.

Test Sector	Noise Reduction CPXI [dB(A)]			
	First Measurement		Second Measurement	
	30 km/h	50 km/h	30 km/h	50 km/h
SEPOR-D-PSMA5 W4 s1	7.8	8.1	7.6	7.9
SEPOR-D-PSMA5 W4 s2	9.7	10.0	8.4	8.8

**Table 9:** Noise reduction of poroelastic mixture placed at the Galaktyczna long test section.

Test Section	Noise Reduction CPXI [dB(A)]					
	First Measurement			Second Measurement		
	50 km/h	80 km/h	110 km/h	50 km/h	80 km/h	110 km/h
SEPOR-G-PSMA5 W4 s1	5.7	5.6	5.2	5.2	4.8	4.2
SEPOR-G-PSMA5 W4 s2	4.6	4.2	3.3	3.7	3.1	2.4

**Table 10:** Noise reduction of poroelastic mixtures on the Kartoszyno long test section.

Test Sector	Noise Reduction CPXI [dB(A)]					
	First Measurement			Second Measurement		
	30 km/h	50 km/h	80 km/h	30 km/h	50 km/h	80 km/h
SEPOR-K-PSMA8 W2 DB	6.3	5.5	6.1	5.7	6.2	6.6
SEPOR-K-PSMA8 W2 PB	7.9	7.8	8.3	8.3	8.5	8.9
SEPOR-K-PSMA5 W6 DB	6.3	6.2	6.5	5.9	6.0	7.5
SEPOR-K-PSMA5 W6 PB	7.8	8.0	8.0	8.3	9.0	9.7
	**Third Measurement**			**Fourth Measurement**		
	**30 km/h**	**50 km/h**	**80 km/h**	**30 km/h**	**50 km/h**	**80 km/h**
SEPOR-K-PSMA8 W2 DB	4.5	3.8	4.4	6.0	4.6	4.6
SEPOR-K-PSMA8 W2 PB	5.9	5.3	5.8	7.1	5.7	5.9
SEPOR-K-PSMA5 W6 DB	4.6	4.0	5.0	7.5	6.0	7.0
SEPOR-K-PSMA5 W6 PB	5.8	5.4	6.2	7.4	5.4	6.8

**Table 11:** Differences in noise levels between SMA11 and PSMA8/PSMA5 pavements.

Speed	Measurement Session	Difference in Noise Levels on SMA11 Pavement and the Poroelastic Pavement [dB]	
		PSMA8	PSMA5
50 km/h	I	5.0	6.2
	II	6.9	8.5
	III	7.7	8.1
	IV	8.4	8.0
80 km/h	I	5.1	6.6
	II	7.8	9.4
	III	8.3	9.4
	IV	8.9	8.1

## Data Availability

Not applicable.
